# Supplementary material for: Structural basis for transthiolation intermediates in the ubiquitin pathway
Source: Nature. 2024 Aug 14;633(8028):216–23. doi: 10.1038/s41586-024-07828-9 (PMC11374688; doi:10.1038/s41586-024-07828-9)
Supplement: Supplementary file 1 — This file contains Supplementary Figs. 1–5 and full descriptions for the supplementary figures, table and videos. [file 41586_2024_7828_MOESM1_ESM.pdf]

nature portfolio

https://doi.org/10.1038/s41586-024-07828-9

Supplementary information

Structural basis for transthiolation intermediates in the ubiquitin pathway

In the format provided by the authors and unedited

SI Guide Table of Contents

Supplementary Table Legends

Supplementary Table 1…………………………………………………………...…page 3

Supplementary Figure Legends

Supplementary Figure 1...………………………………………………………...…page 3

Supplementary Figure 2...………………………………………………………...…page 3

Supplementary Figure 3...………………………………………………………...…page 3

Supplementary Figure 4...………………………………………………………...…page 4

Supplementary Figure 5...………………………………………………………...…page 4

Supplementary Video Legends

Supplementary Video 1...………………………………………………………...…page 5

Supplementary Video 2...………………………………………………………...…page 5

Supplementary Figures……………………………………………………………..page 6

Supplementary Table Legends

***Supplementary Table 1* | Statistics and data collection information for cryo-EM reconstructions and molecular models**. Excel spreadsheet supplied separately (.xlsx). Excel file is broken down into two sheets by samples with one sheet for singly and doubly-loaded E1-Ub-E2 complexes and one sheet for the E2-Ub-E3 complexes.

Supplementary Figure Legends

Supplementary Figure 1 | Raw uncropped gels for image shown in Figure 1c. Portable Document Format (.pdf). SDS-PAGE gels separating reactants in reactions of Ub-PSAN with E2 (containing C-terminal His_6-tag) (**a**) and E2-Ub vinyl thioether (E2 with His_6-tag cleaved) with E1 (**b**) and $E3^{HECT}$ (**c**). Dashed boxes indicate regions of the gel shown in Figure 1c, molecular weight markers are indicated and labeled on the left.

Supplementary Figure 2 | Particle orientation information and FSC curves for cryo-EM reconstructions. Portable Document Format (.pdf). Names of reconstructions and corresponding particle counts (shown in brackets) (left), viewing direction distribution as Euler angle distribution plot (middle), and Fourier Shell Correlation (FSC) between half maps and between the refined model and full map (right) for singly and doubly-loaded E1-Ub-E2 complexes (**a**) and for E2-Ub-E3 complexes (**b**).

Supplementary Figure 3 | Raw uncropped gels shown in Figure 3g and quantified in Figure 3h. Portable Document Format (.pdf). **a**, SDS-PAGE analysis of molecular weight markers run with the same conditions for (**b**) purified E1~Ub thioester featuring fluorescently labeled Ub. **c**, SDS-PAGE analysis of molecular weight markers run alongside E1~Ub, E2~Ub and Ub featuring fluorescent Ub run under similar conditions as (**d**) showing assays monitoring transfer of fluorescent Ub from purified E1~Ub thioester to E2 under indicated conditions (three replicates shown). Molecular weight standards were not run on gels in panels **b** and **d** but are labeled in panels **b** and **d** based on their positions in gels depicted in panels **a** and **c**. Gel in panel

b is representative of three independent preparations, and gels in panel **d** show three independent replicates. Green triangle indicates fluorescent ubiquitin.

Supplementary Figure 4 | Raw uncropped gels for data quantified in Figures 4g and 5d. Portable Document Format (.pdf). **a**, SDS-PAGE analysis of molecular weight markers run with the same conditions for panels **b-f** scanned for fluorescently labeled Ub with E1~Ub, E3~Ub and E2~Ub and Ub indicated on the right. **b**, Wild-type Ub and E2 variants were used to generate E2~Ub(T) and wild-type or mutant variant $E3^{HECT}$ was added at the start of the chase and reaction was quenched at the indicated time points in a time course. **c**, Pulse chase assay monitoring transfer of fluorescently labeled Ub from E2~Ub(T) thioester to $E3^{HECT}$ performed in technical triplicates. Wild-type Ub and E2 were used to generate E2~Ub(T) and wild-type or mutant variant $E3^{HECT}$ was added at the start of the chase. Reactions were quenched at 15 second time point. **d**, Wild-type E2 and wild-type and indicated mutant variants Ub were used to generate E2~Ub(T). Wild-type $E3^{HECT}$ was added at the start of the chase. The reactions were quenched at 15 second time point and performed in technical triplicates. **e**, Pulse chase assays monitoring transfer of fluorescent Ub from E2~Ub(T) thioester to $E3^{HECT}$ performed in technical triplicates with wild-type and indicated mutant variants of E2 and wild-type Ub were used to generate E2~Ub(T). Wild-type $E3^{HECT}$ was added to start the chase and the reactions were quenched at 15 second time point. **f**, Wild-type or indicated mutant variant $E3^{HECT}$ was added to wild-type or indicated mutant E2 at the start of the chase and the reactions were quenched at 15 second time point. Molecular weight standards were not run on gels but are labeled on the left for panels **e-f** based on their positions in the gel for panel **a**. Green triangle indicates fluorescent ubiquitin.

Supplementary Figure 5 | Raw uncropped gels for data quantified in Figure 5c. Portable Document Format (.pdf). **a**, SDS-PAGE analysis of molecular weight markers run with the same conditions for panel **b** scanned for fluorescence after staining with Flamingo staining with E1~Ub, E3~Ub and E2~Ub and Ub indicated on the right. **b**, SDS-PAGE gels to visualize E1-E2 Ub thioester transfer assays performed under multiple turnover conditions performed in technical triplicates as described in Methods. E2 amino acid substitutions indicated above each gel with E2, E2~Ub(T), E1 and E1~Ub(T) positions on the right. Gels imaged as in panel **a**. Molecular

weight standards were not run on gels in panel **b** but are labeled on the left based on positions in the gel for panel **a**. Gel in panel **a** is representative of three independent preparations, and gels in panel **b** show three independent replicates.

Supplementary Video Legends (Videos supplied separately)

Supplementary Video 1 | Side-by-side comparison of E1-Ub-E2 reconstructions. MP4 File (.mp4). Side-by-side views of reconstructions and models for singly and doubly loaded E1 complexes to facilitate comparison of conformational changes that accompany transfer of ubiquitin from E1 to E2, and for doubly loaded E1, how conformational changes for ubiquitin undergoing transthiolation – Ub(T)- are coupled to the chemical steps of ubiquitin adenylation – Ub(A) – and pyrophosphate release. Maps and models are low pass filtered as noted to enable visualization of the less stable Ub(T) intermediates in this process. Isosurface levels contoured at 0.25-0.28 (low pass filtered maps) (**i**); 0.41, 0.43, 0.41, 0.52, 0.44 for clusters 1, 2, 3, 4, 5 in singly-loaded complex and 0.66, 0.59, 0.58, 0.48, 0.46 for clusters 1, 2, 3, 4, 5 in doubly-loaded complex ((T) site); 1.1, 1.1, 1.0, 1.0, 0.9 for clusters 1, 2, 3, 4, 5 in singly-loaded complex and 1.2, 1.2, 1.2, 1.2, 1.2 for clusters 1, 2, 3, 4, 5 in doubly-loaded complex ((A) site).

Supplementary Video 2 | Comparison of E2-Ub-E3^{HECT} reconstructions. MP4 File (.mp4). Comparison of reconstructions for E2-Ub-E3^{HECT} in States 1-7 (unsharpened maps) showing conformational changes in Ub(T) and E2 as Ub(T) moves from E2 to E3. Zoom in to show side chains involved in contacts to the E2-Ub-E3^{HECT} transthiolation active site to highlight changes in E2 loop and the Ub(T) C-terminal tail between State 1 and State 2. Isosurface levels contoured at 0.47-0.52.

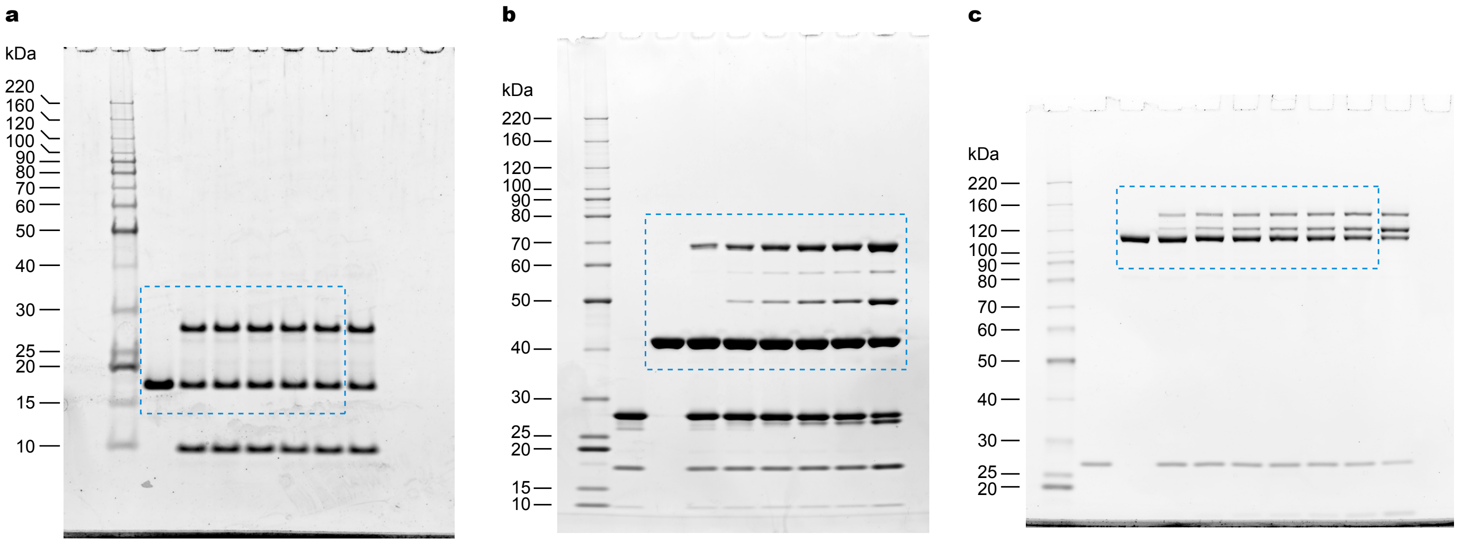

Supplementary Figure 1 | Raw uncropped gels for image shown in Figure 1c.

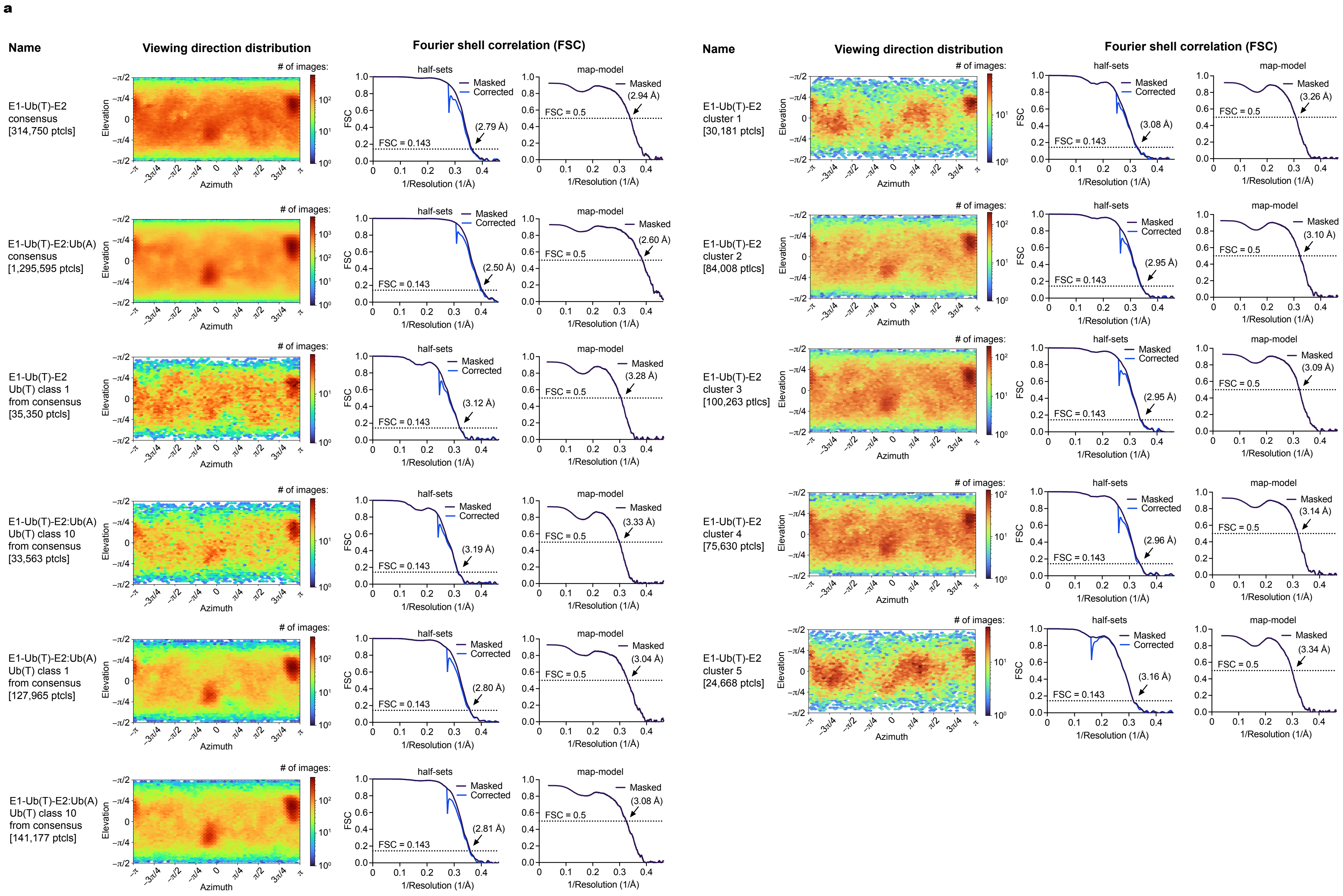

Supplementary Figure 2 | Particle orientation information and FSC curves for cryo-EM reconstructions.

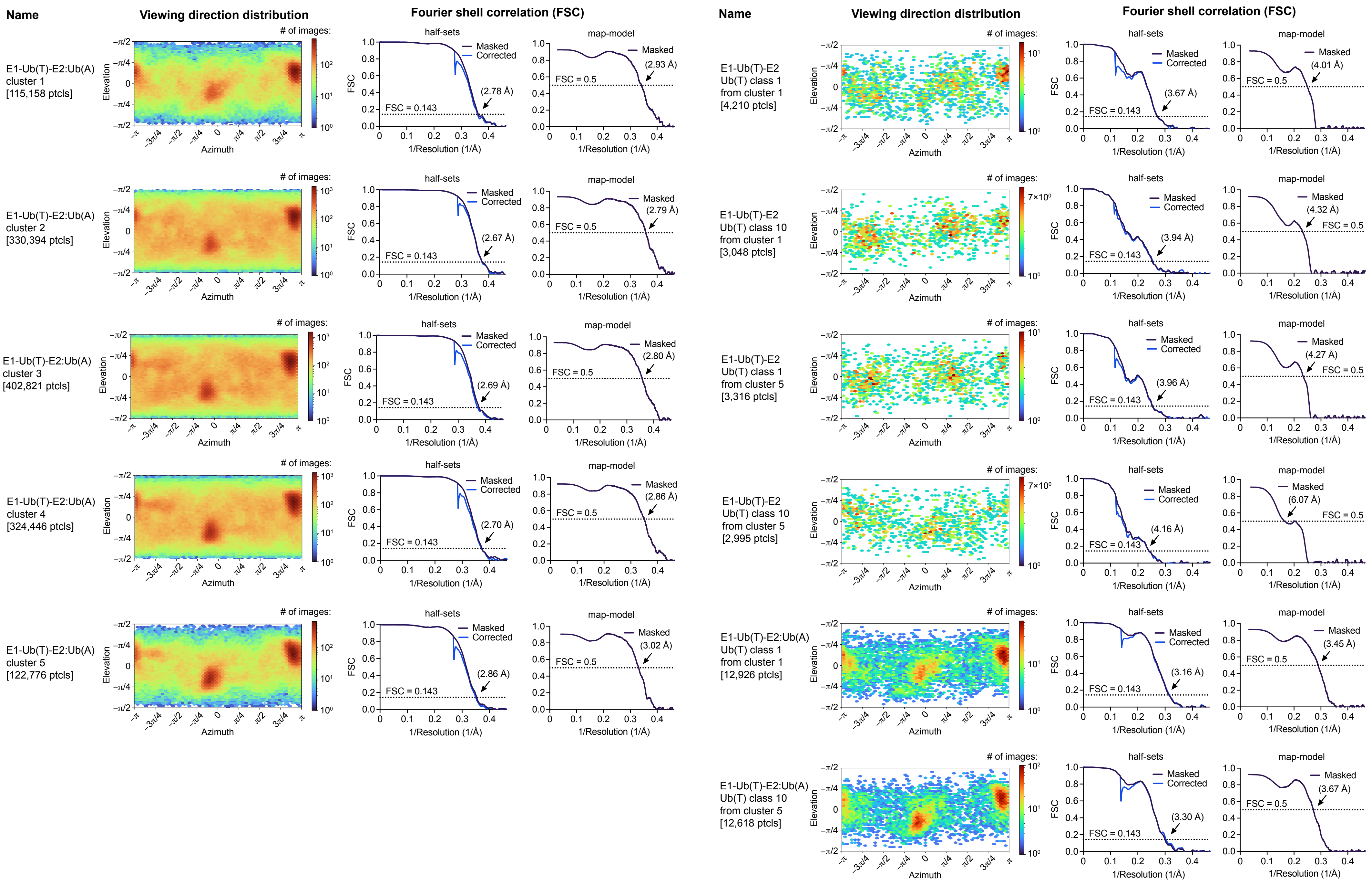

Supplementary Figure 2 (continued) | Particle orientation information and FSC curves for cryo-EM reconstructions.

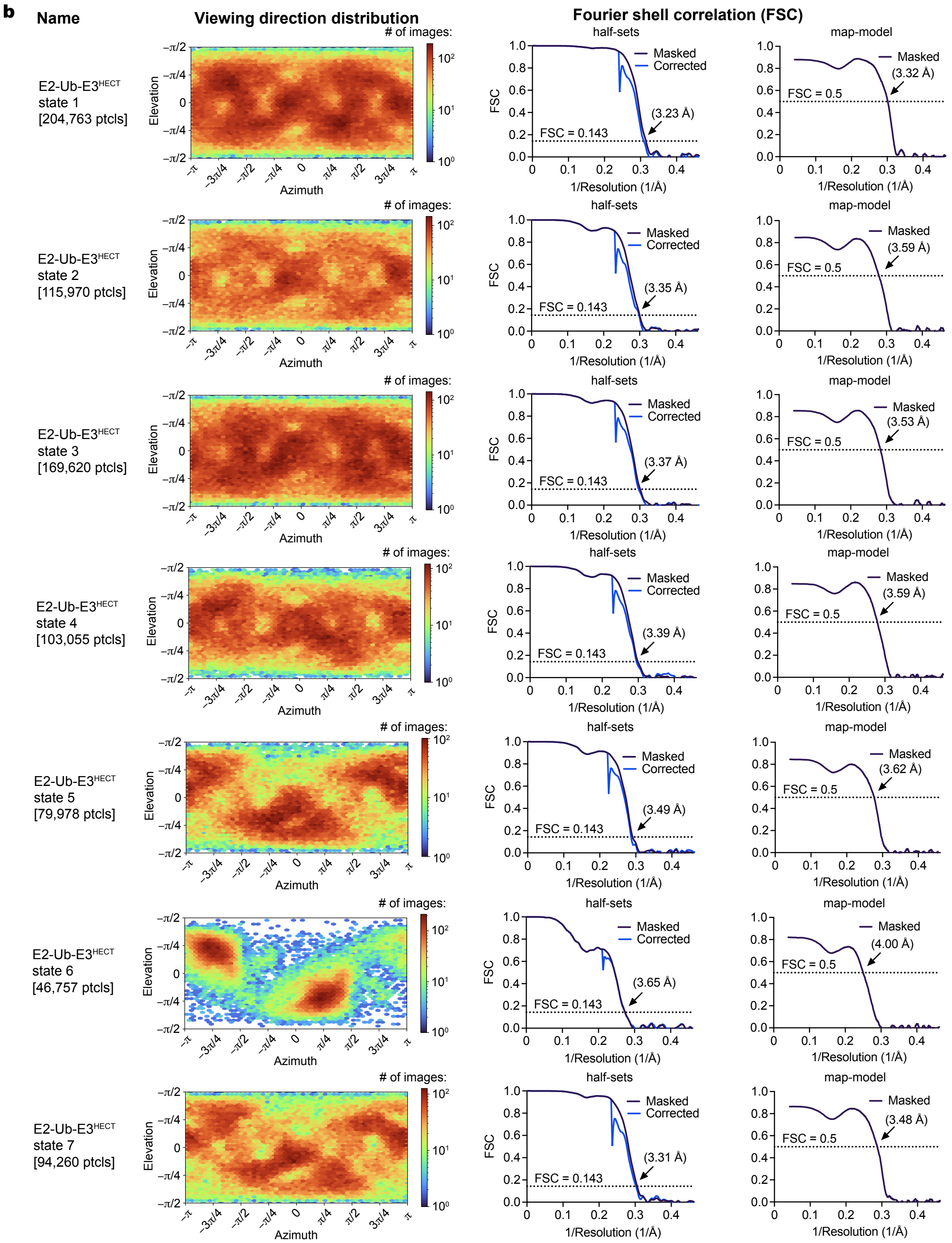

Supplementary Figure 2 (continued) | Particle orientation information and FSC curves for cryo-EM reconstructions.

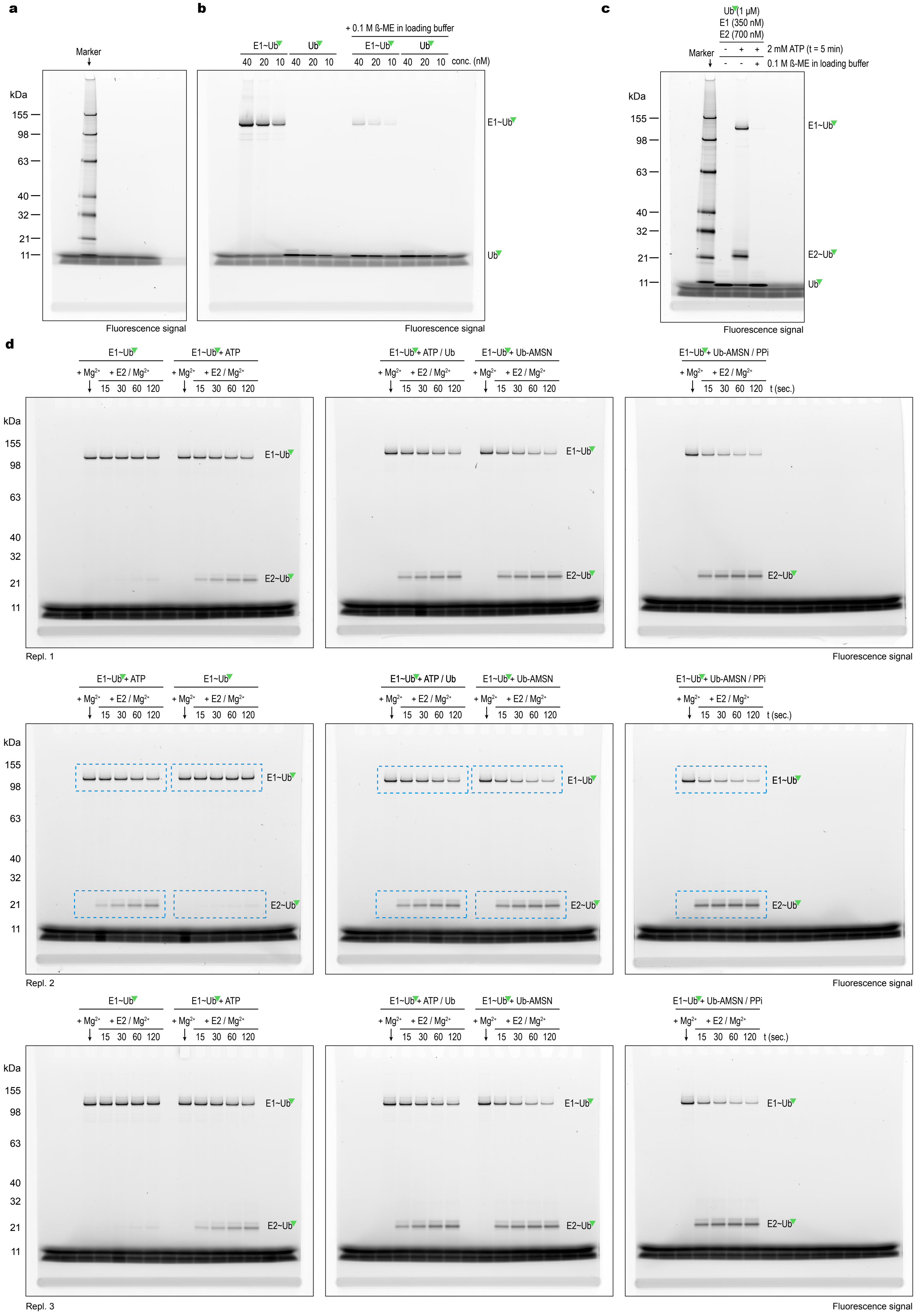

Supplementary Figure 3 | Raw uncropped gels shown in Figure 3g and quantified in Figure 3h.

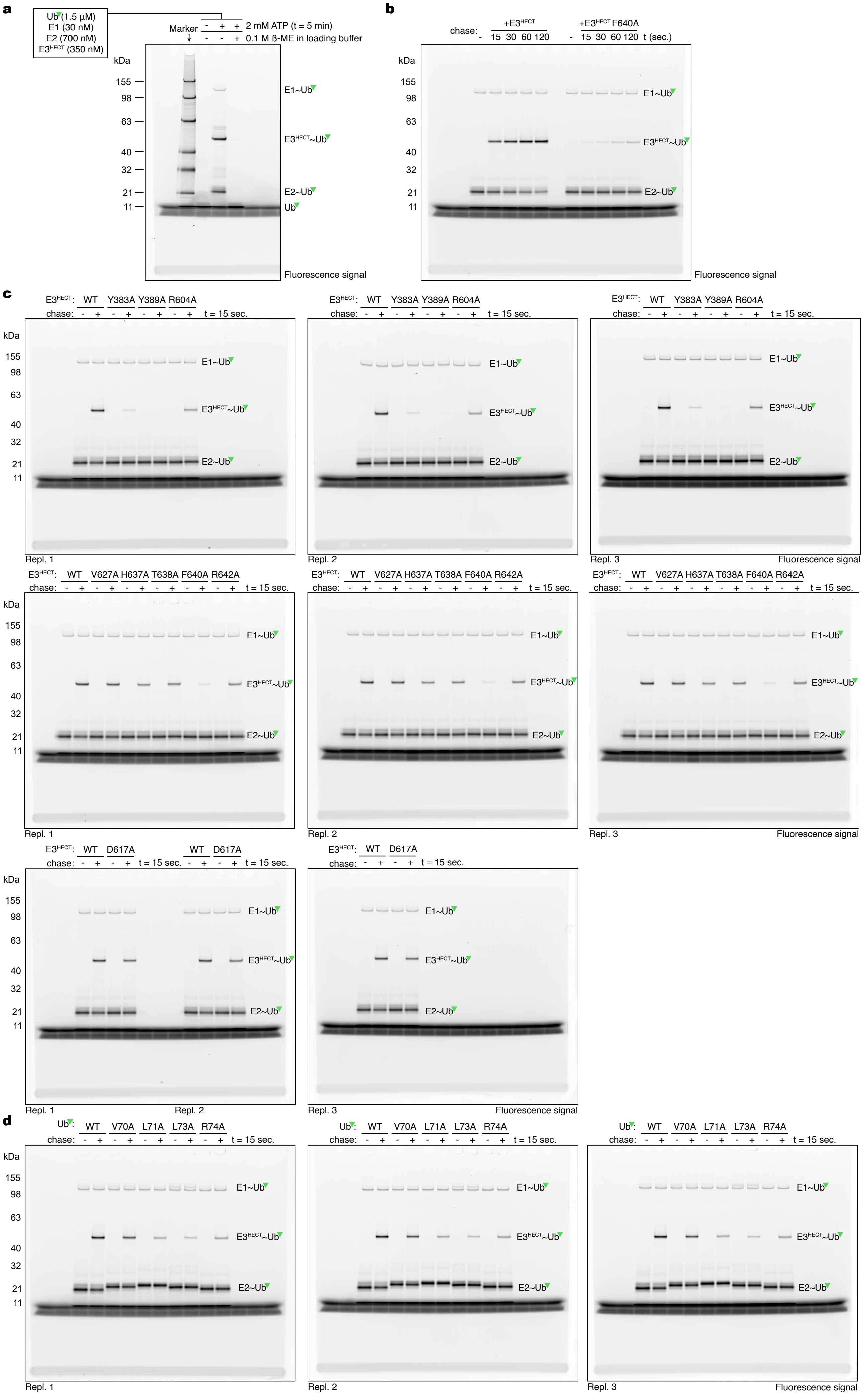

Supplementary Figure 4 | Raw uncropped gels for data quantified in Figures 4g and 5d.

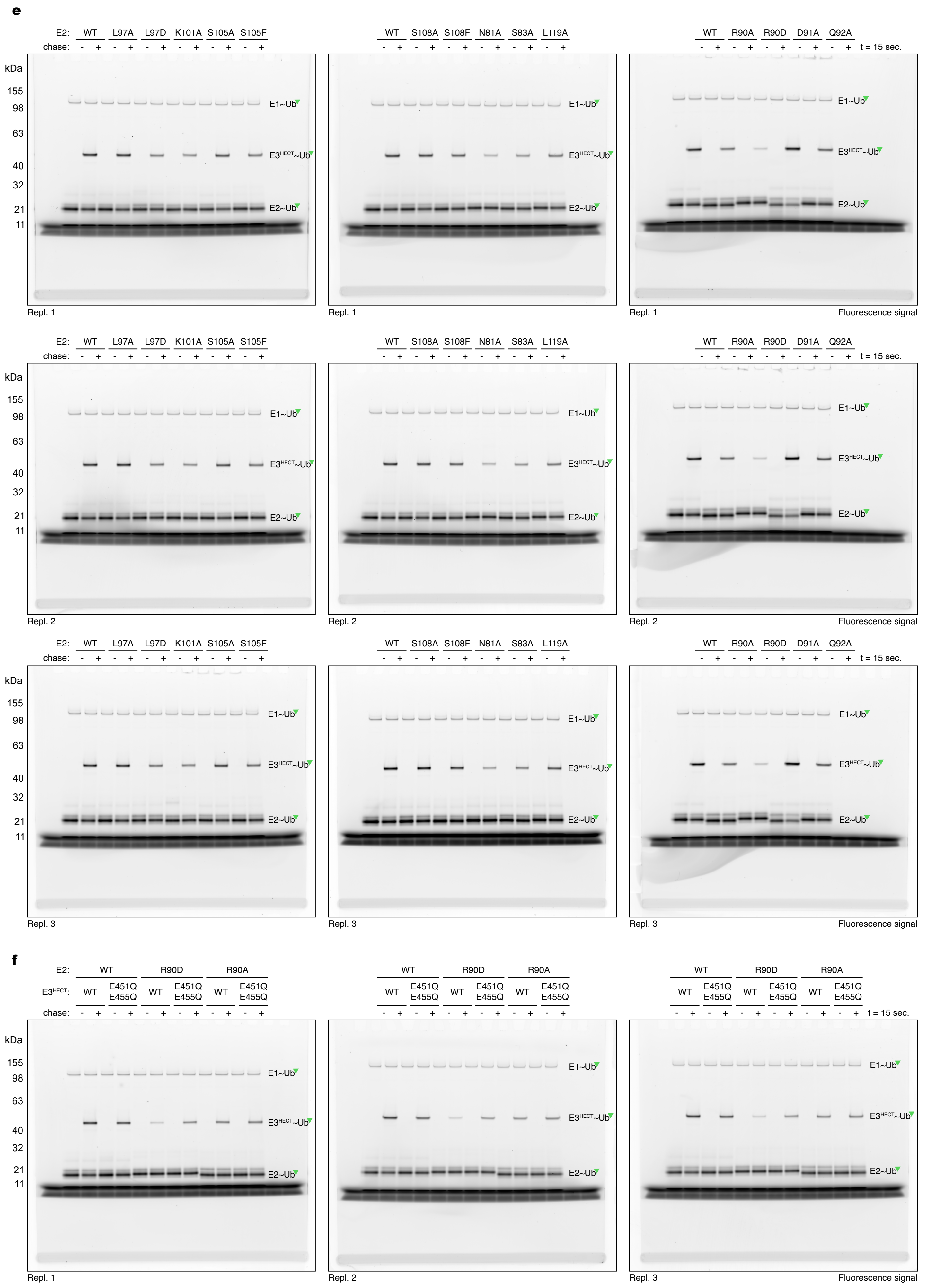

Supplementary Figure 4 (continued) | Raw uncropped gels for data quantified in Figures 4g and 5d.

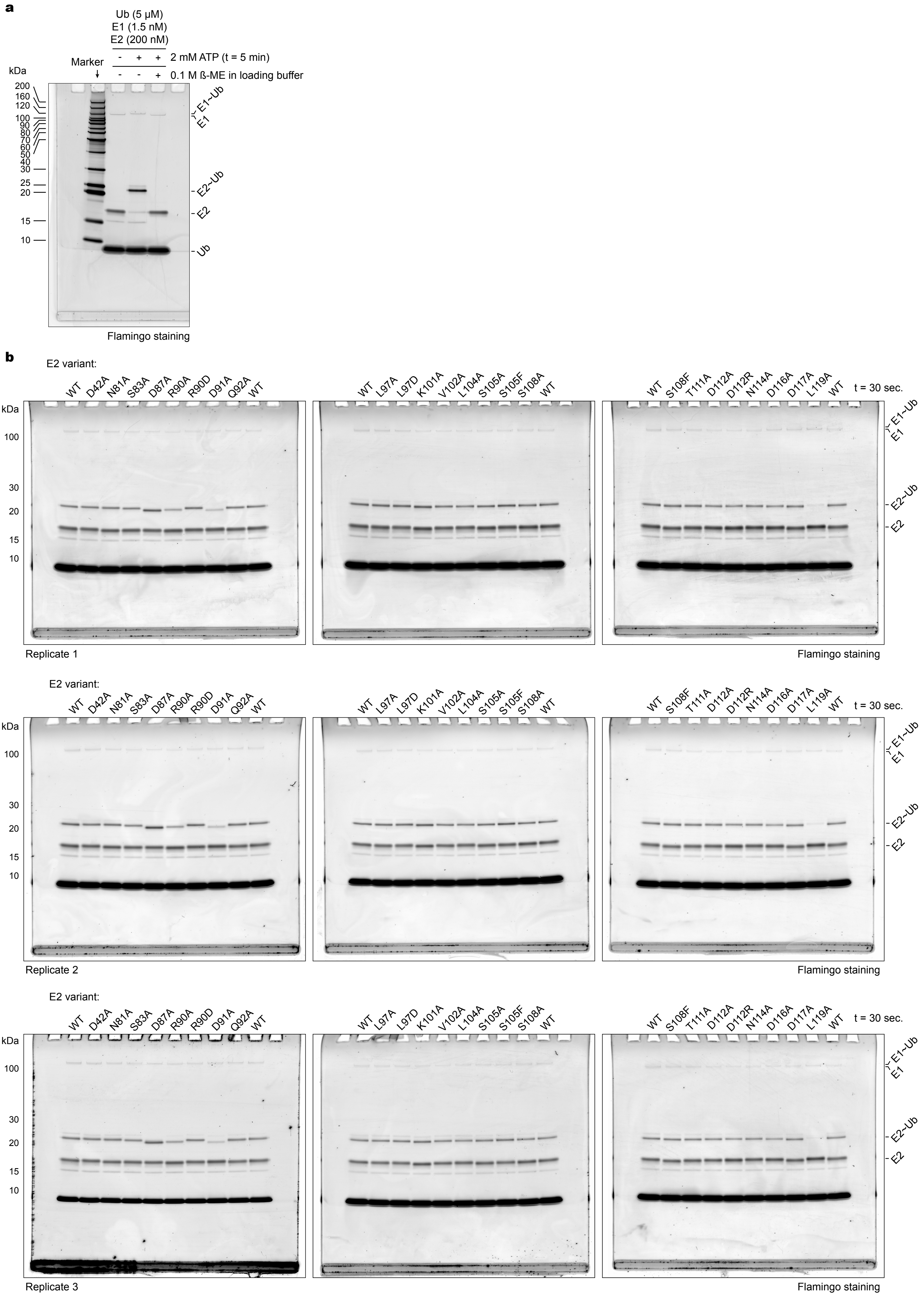

Supplementary Figure 5 | Raw uncropped gels for data quantified in Figure 5c.
