# Supplementary material for: Structural basis for transthiolation intermediates in the ubiquitin pathway
Source: Nature. 2024 Aug 14;633(8028):216–23. doi: 10.1038/s41586-024-07828-9 (PMC11374688; doi:10.1038/s41586-024-07828-9)
Supplement: Supplementary file 2 — Reporting Summary [file 41586_2024_7828_MOESM2_ESM.pdf]

nature portfolio

Corresponding author(s): Christopher D. Lima
Derek S. Tan

Last updated by author(s): May 24, 2024

Reporting Summary

Nature Portfolio wishes to improve the reproducibility of the work that we publish. This form provides structure for consistency and transparency in reporting. For further information on Nature Portfolio policies, see our Editorial Policies and the Editorial Policy Checklist.

Statistics

For all statistical analyses, confirm that the following items are present in the figure legend, table legend, main text, or Methods section.

n/a	Confirmed	
☐	☒	The exact sample size (*n*) for each experimental group/condition, given as a discrete number and unit of measurement
☐	☒	A statement on whether measurements were taken from distinct samples or whether the same sample was measured repeatedly
☐	☒	The statistical test(s) used AND whether they are one- or two-sided<br>*Only common tests should be described solely by name; describe more complex techniques in the Methods section.*
☒	☐	A description of all covariates tested
☐	☒	A description of any assumptions or corrections, such as tests of normality and adjustment for multiple comparisons
☐	☒	A full description of the statistical parameters including central tendency (e.g. means) or other basic estimates (e.g. regression coefficient) AND variation (e.g. standard deviation) or associated estimates of uncertainty (e.g. confidence intervals)
☐	☒	For null hypothesis testing, the test statistic (e.g. *F*, *t*, *r*) with confidence intervals, effect sizes, degrees of freedom and *P* value noted<br>*Give P values as exact values whenever suitable.*
☒	☐	For Bayesian analysis, information on the choice of priors and Markov chain Monte Carlo settings
☒	☐	For hierarchical and complex designs, identification of the appropriate level for tests and full reporting of outcomes
☒	☐	Estimates of effect sizes (e.g. Cohen's *d*, Pearson's *r*), indicating how they were calculated

Our web collection on statistics for biologists contains articles on many of the points above.

Software and code

Policy information about availability of computer code

Data collection	Cryo-EM data collection was performed in SerialEM 3.9.0.
Data analysis	Cryo-EM data were processed using Relion 3.1.3 , MotionCor2 1.3.2, gctf 1.06, Topaz 0.2.5, cryoSPARC versions 3.3.1 and 4.0.2.<br>Molecular models were docked in UCSF Chimera 1.15 and build and refined using Coot 0.9.8 and PHENIX 1.20.1-4487, and analyzed using MolProbity which is integrated into PHENIX 1.20.1-4487.<br>Structure representations were generated using UCSF ChimeraX 1.7.1. 2D slice views of EM-maps were visualized using IMOD 4.11.<br>Densitometric analysis and visualisation of gel scans was performed in ImageQuant TL versions 8.2.0 and 10.2. Calculations were performed in Excel 16.57. Statistical analyses and plotting of the data was performed in Prism 10.2.0. Sequence alignment logos generated using WebLogo 2.8.2.

For manuscripts utilizing custom algorithms or software that are central to the research but not yet described in published literature, software must be made available to editors and reviewers. We strongly encourage code deposition in a community repository (e.g. GitHub). See the Nature Portfolio guidelines for submitting code & software for further information.

Data

Policy information about availability of data

All manuscripts must include a data availability statement. This statement should provide the following information, where applicable:

- Accession codes, unique identifiers, or web links for publicly available datasets
- A description of any restrictions on data availability
- For clinical datasets or third party data, please ensure that the statement adheres to our policy

Cryo-EM reconstructions and coordinates are deposited and available at the Electron Microscopy Data Bank (emdataresource.org) and PDB (rcsb.org), respectively. For singly loaded E1-Ub(T)-E2, cryo-EM coordinates and maps are deposited under accession codes 9B5M and EMD-44217 (consensus), 9B5N and EMD-44218 (consensus, state 1), 9B5O and EMD-44219 (consensus, state 10), 9B5P and EMD-44220 (cluster 1), 9B5U and EMD-44225 (cluster 1, state 1), 9B5V and EMD-44226 (cluster 1, state 10), 9B5Q and EMD-44221 (cluster 2), 9B5R and EMD-44222 (cluster 3), 9B5S and EMD-44223 (cluster 4), 9B5T and EMD-44224 (cluster 5), 9B5W and EMD-44227 (cluster 5, state 1) and 9B5X and EMD-44228 (cluster 5, state 10) with cryo-EM maps for 3D classes representing states 1-10 as additional maps under accession codes for consensus (10 maps) and for clusters 1-5 (50 maps, 10 per cluster). For doubly loaded E1-Ub(T)-E2 with Ub(A), cryo-EM coordinates and maps are deposited under accession codes 9B5C and EMD-44207 (consensus), 9B5D and EMD-44208 (consensus, state 1), 9B5E and EMD-44209 (consensus, state 10), 9B5F and EMD-44210 (cluster 1), 9B5K and EMD-44215 (cluster 1, state 1), 9B5G and EMD-44211 (cluster 2), 9B5H and EMD-44212 (cluster 3), 9B5I and EMD-44213 (cluster 4), 9B5J and EMD-44214 (cluster 5), and 9B5L and EMD-44216 (cluster 5, state 10) with cryo-EM maps for 3D classes representing states 1-10 as additional maps under accession codes for consensus (10 maps) and for clusters 1-5 (50 maps, 10 per cluster). For E2-Ub(T)-E3, cryo-EM coordinates and maps are deposited under accession codes 9B55 and EMD-44200 (state 1), 9B56 and EMD-44201 (state 2), 9B57 and EMD-44202 (state 3), 9B58 and EMD-44203 (state 4), 9B59 and EMD-44204 (state 5), 9B5A and EMD-44205 (state 6), and 9B5B and EMD-44206 (state 7). The atomic coordinates of previously published structures of ubiquitin E1 crosslinked toE2 (Ubc4) with ubiquitin (4II2), ubiquitin E1 bound to ubiquitin-AMSN (6o82) and E2~ubiquitin~HECT (3jw0) were used in this study. All relevant data are included in the manuscript. Supplemental Data Figures contain uncropped gel images for all replicates. There are no restrictions on data availability. Source data are provided with this paper.

Research involving human participants, their data, or biological material

Policy information about studies with human participants or human data. See also policy information about sex, gender (identity/presentation), and sexual orientation and race, ethnicity and racism.

Reporting on sex and gender	n/a
Reporting on race, ethnicity, or other socially relevant groupings	n/a
Population characteristics	n/a
Recruitment	n/a
Ethics oversight	n/a

Note that full information on the approval of the study protocol must also be provided in the manuscript.

Field-specific reporting

Please select the one below that is the best fit for your research. If you are not sure, read the appropriate sections before making your selection.

☒ Life sciences ☐ Behavioural & social sciences ☐ Ecological, evolutionary & environmental sciences

For a reference copy of the document with all sections, see nature.com/documents/nr-reporting-summary-flat.pdf

Life sciences study design

All studies must disclose on these points even when the disclosure is negative.

Sample size	Sample sizes were not predetermined. Cryo-EM sample size was determined by the available microscope time. The number of images collected is indicated in Extended Data Figures 2 and 8. Biochemical sample sizes were not predetermined. Biochemical sample size was determined after three independent replicates and evaluation of statistical significance to ensure reproducibility.
Data exclusions	Cryo-EM images were excluded from the data sets when they showed evidence of crystalline ice, estimated resolution limits worse than 4.5 Å or poor CTF fit. Particles belonging to bad classes were excluded during 2D classifications if their 2D class averages represented noise. The selection of particles are shown in Extended Data Figures 2 and 8.
Replication	Cryo-EM: Each condition (E1-Ub(T)-E2 and E2-Ub(T)-E3) was imaged from two grids.<br>Biochemical analysis: Each condition was examined on at least two different days using three preparations of protein with three independent replicates. All attempts at replication were successful. All biochemical experiments were performed in triplicate, as indicated in the figure legends. For a detailed description, please see the 'Statistics and reproducibility' section in the methods.

Randomization	For cryo-EM 3D refinements, all particles are randomly split into two groups and two independent reconstructions are generated. The two groups are refined independently and the Fourier Shell Correlation (FSC) between the independent reconstructions is computed according to gold-standard procedure. Randomization is not applicable to other experiments described here.
Blinding	Blinding was not performed for cryo-EM image analysis as it requires manual evaluation at the step of image processing to ensure high-quality reconstructions. Blinding is not relevant to biochemical experiments because no group allocation was involved. Data were analyzed using unbiased methods.

Reporting for specific materials, systems and methods

We require information from authors about some types of materials, experimental systems and methods used in many studies. Here, indicate whether each material, system or method listed is relevant to your study. If you are not sure if a list item applies to your research, read the appropriate section before selecting a response.

Materials & experimental systems

n/a	Involved in the study	
☒	☐	Antibodies
☒	☐	Eukaryotic cell lines
☒	☐	Palaeontology and archaeology
☒	☐	Animals and other organisms
☒	☐	Clinical data
☒	☐	Dual use research of concern
☒	☐	Plants

Methods

n/a	Involved in the study	
☒	☐	ChIP-seq
☒	☐	Flow cytometry
☒	☐	MRI-based neuroimaging

Plants

Seed stocks	n/a
Novel plant genotypes	n/a
Authentication	n/a
